# Comparison of the phlebotomine (Diptera: Psychodidae) fauna of urban, transitional, and wild areas in northern Minas Gerais, Brazil

**DOI:** 10.1186/s13071-015-1003-2

**Published:** 2015-08-19

**Authors:** Cristiani de Castilho Sanguinette, Danyele Franca da Silva, Rodolfo German Antonelli Vidal Stumpp, Felipe Dutra Rego, Gabriel Barbosa Tonelli, Aline Tanure, Célia Maria Ferreira Gontijo, José Dilermando Andrade Filho

**Affiliations:** Leishmaniases Research Group, Phlebotomine Collection, National and International Reference Center for Phlebotomines, René Rachou Institute, Fiocruz, Av. Augusto de Lima 1715, 30190-002 Belo Horizonte, MG Brasil; Universidade Federal de Minas Gerais (UFMG), Belo Horizonte, Brasil

**Keywords:** Phlebotominae, Fauna, Vector ecology, Leishmaniasis

## Abstract

**Background:**

Phlebotomines are directly related to the study of leishmaniases, and so the study of their distribution plays an important role in the epidemiology of these diseases. Collections of phlebotomines were made with the intent of comparing the distribution, richness, diversity, and abundance of species in three distinct environments in an area endemic for tegumentary and visceral leishmaniasis in Minas Gerais State, Brazil.

**Methods:**

Phlebotomines were collected with automatic light traps in urban, transitional, and wild areas from March 2013 to February 2014 in the district of Barra do Guaicuí, municipality of Várzea da Palma, Minas Gerais. The distribution patterns of these species of insects, as well as species richness, evenness, and abundance among the different areas, were analyzed.

**Results:**

A total of 3,365 phlebotomines belonging to 15 species were collected. The urban area had the greatest abundance whereas the transitional area had the greatest diversity and evenness of species. *Nyssomyia intermedia* was the most abundant species in the urban area, whereas *Evandromyia evandroi* was the most abundant in the transitional area and *Ev. lenti* in the wild area.

**Conclusion:**

The analysis of our results showed that the distribution of the collected species had distinct profiles between the environments studied. Furthermore our study indicates the potential risk of transmission of leishmaniasis in the urban environment where it was observed had the highest population density and abundance of important vector species of *Leishmania*.

## Background

Environmental changes derived from human actions, such as the rapid process of urbanization observed in emerging countries like Brazil, have changed the ecology of some species of phlebotomines and, consequently, the eco-epidemiology of the leishmaniases. In respect of visceral leishmaniasis these changes lead to a significant impact on the distribution and mortality rates of the disease since the 1980s [1, 2]. Thus, four new patterns have emerged: the transmission of visceral leishmaniasis (VL) in fully urbanized areas; the rapid spread in cities of the Northeast and in various cities in the North, Central-West, and Southeast; an increase in the number of cases in urban areas compared to rural areas; and the emergence of large-scale urban epidemics with cycles of ten years [3].

The increase in the number of cases of tegumentary leishmaniasis (TL) and VL in Minas Gerais in recent years is documented by the System of Information of Disease Notification from 2000 to 2009, who reports that in Minas Gerais there is an average of 390 cases of VL annually [4, 5]. Currently, in Minas Gerais, the cities of Belo Horizonte, Montes Claros, Ribeirão da Neves, Janaúba, Santa Luzia and Paracatu correspond to 56 % of the VL cases reported in the state [6].

The first case of TL in the municipality of Várzea da Palma was reported in 2001 in the district of Barra do Guaicui. Since then, from 2004 to 2013, 202 human cases of autochthonous TL and 37 cases of VL have been reported (Source: Municipal Secretary of Health of Várzea da Palma, MG).

One of the challenges related to the control of leishmaniases is the lack of knowledge about epidemiological conditions, especially regarding the distribution of phlebotomine populations, the behavior and the identification of these species. Thus, this study aims to present the distribution, richness, diversity, and abundance of species of phlebotomines related to urban, transitional, and wild environments in the district of Barra do Guaicuí, an endemic area for TL and VL in Minas Gerais state. Not only can this information provide a more accurate description of the current situation of the sand fly fauna, but it can also be predictive, and thus allowing the establishment of more effective control methods and epidemiological surveillance in the region.

## Methods

### Study area

**Fig. 1:**
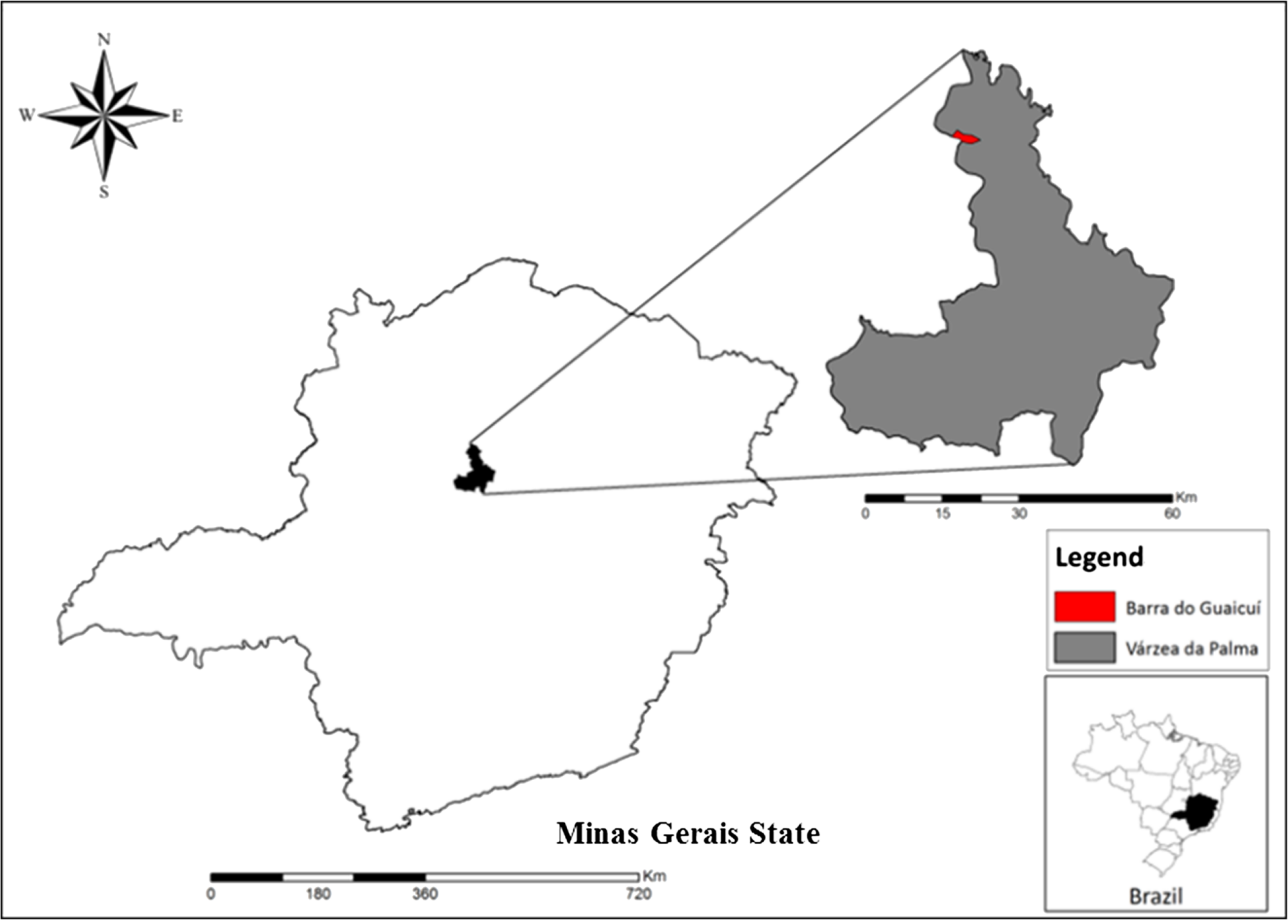
Municipality of Várzea da Palma, showing the district of Barra do Guaicuí, in northern Minas Gerais, Brazil

The study area, Barra do Guaicui (17°12’S and 44°48’W), is a district located in the municipality of Várzea da Palmain the northern region of Minas Gerais, Brazil (Fig 1). According to the Brazilian Institute of Geography and Statistics the municipality is represented by an area of 2,220.279 km^2^ and a population, estimated in 2014, of 38,213 inhabitants [7].

The municipality varies in elevation between 480 and 800 m, with a mountain range that connects with the Serra do Cabral [8]. According to Köppen *et al*., the climate is classified as tropical savanna with a dry season in the winter [9]. Várzea da Palma is situated in the savannah (cerrado biome) although some open areas east of Várzea da Palma show a caatinga biome influence. Small fragments of forest in various states of succession are also present forming a mosaic of vegetation that varies in complexity from shrubs to very dense forest [10]. The prevalent phytophysiognomies are open fields, cerrado *sensu stricto* and seasonal deciduous montane forest, with the best preserved areas of vegetation being found at higher elevations [8].

### Collection of phlebotomine sand flies

The urban area (A) is located in the center of the district of Barra do Guaicuí and has a population of 3,000 inhabitants, many brickwork buildings and some unoccupied areas that have become overgrown. The native vegetation, cerrado *sensu stricto,* is practically nonexistent and is replaced by other types of plants, principally mangos. The cerrado *sensu stricto* is characterized by the presence of low, twisted trees with irregular branches. The bushes and subshrubs are spread, with some species with perennial underground organs which allow regrowth after cutting or burning. The trunks of woody plants in general have shells with thick cork and rigid leathery leaves. These characters suggest adaptation to drought conditions. The organic matter content varies from medium to low [11].

The traps were exposed in the peridomiciliary areas and, when present, in chicken house, pig pen or kennel. These areas experiences flooding during the rainy season due to the close proximity of the Velhas River.

The transitional area (T1), or hemi-synanthropic area, is located in a remote village with a few nearby buildings. The sampled houses were at a distance of 10 meters from the forest and with the presence of animals such as horse, goat, chicken and dog, being created free. The native vegetation, seasonal deciduous montane forest, is present despite being widely used by residents as a source of firewood. Some nearby areas are used as pasture.

The wild area was sampled at three points (T2, T3 and T4) that were away from human habitations and feature native vegetation of seasonal deciduous preserved montane forest. This kind of forest has as a main feature two well-marked seasons, one rainy and the other a long drought in which more than half of the vegetation loses its leaves, allowing the presence of organic matter in the soil.

**Fig. 2:**
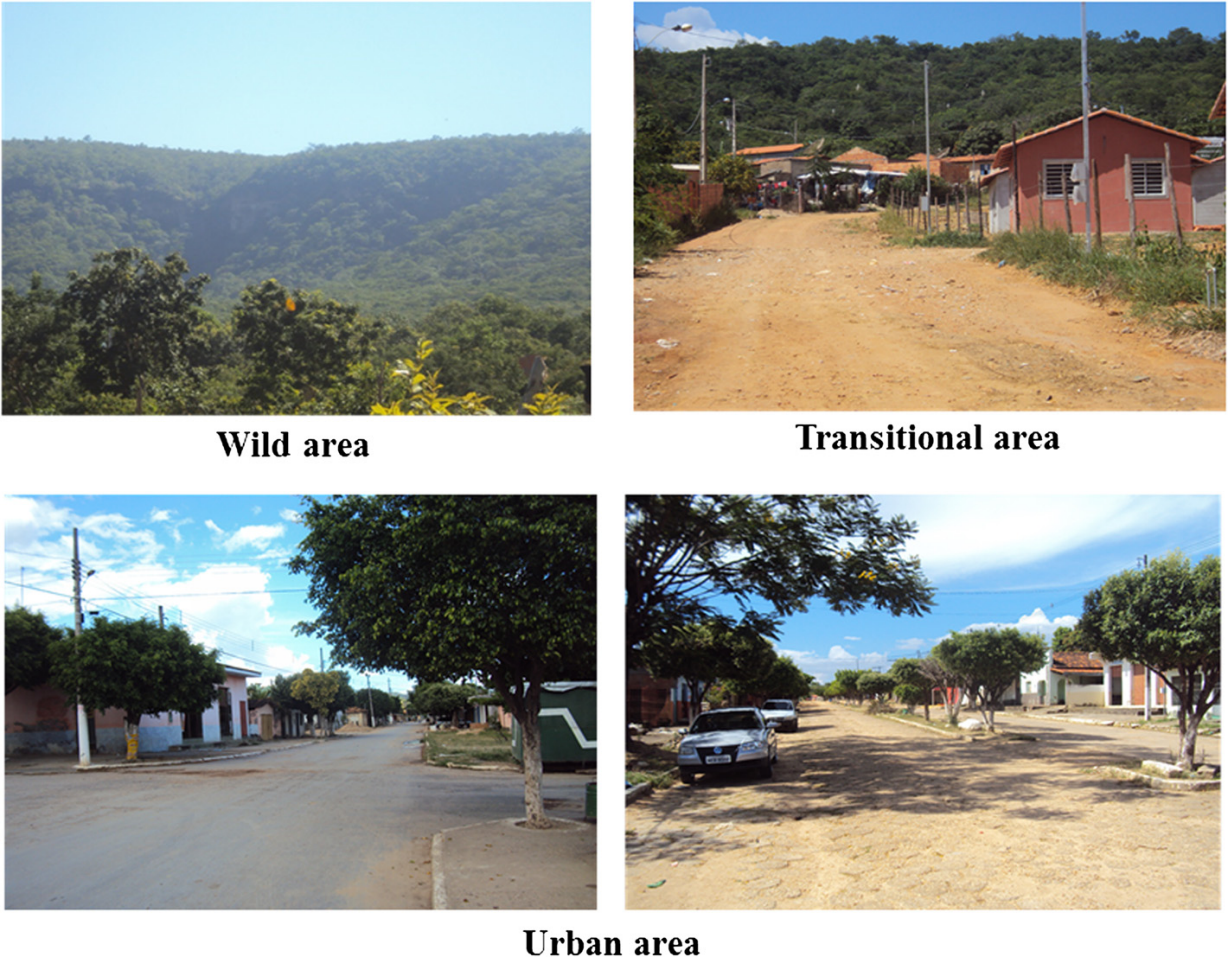
Localities where phlebotomines were collected in 2013 and 2014 in the district of Barra do Guaicui, Minas Gerais, Brazil

Phlebotomines were collected from March 2013 to February 2014 using HP-model automatic light traps [12]. A total of ten light traps were installed monthly: two in the transitional area (forest edge at a distance of 100 meters from the house); six in the wild area with two at each of the three sample points; and two in the peridomicile area of houses in the urban area (Fig 2). The traps were set during three consecutive nights from 18:00 to 6:00 hours, totaling 36 hours of sampling effort per night for each trap. In all study sites traps were installed about 1 meter from the ground.

The traps were removed after each night so that sorting and sexing of phlebotomines could be performed. All specimens were prepared, mounted, and identified according to the routine procedures adopted by the National and International Reference Center for Phlebotomines (CRNIF) of the René Rachou/FIOCRUZ Research Center [13].

The collected phlebotomines were identified to species level using light microscopy. The classification followed the proposal of Galati [14]. The abbreviation of the generic names in this study follows the proposal of Marcondes [15].

The different number of traps in each area resulted in different sampling efforts. These differences were mitigated by multiplying the total number of traps in an area by the number of days the traps were working. The sampling effort was used to calculate capture success, which indicates the actual efficiency of a type of trap in an area. Capture success was calculated using the total number of individuals divided by the sampling effort.

For the evaluation of the most abundant species in each area, the Index of Species Abundance (ISA) was converted into a scale of zero to one by the Standardized Index of Species Abundance (SISA). In this index, value one corresponds to the most abundant species [16]. Richness is considered as the total number of species, and abundance is considered the total number of individuals collected. The diversity and evenness of species of phlebotomines from the different areas were estimated by using the diversity index of Shannon-Wiener (H’) and Pielou (J), respectively [17]. For the species most prevalent in the study, abundances were compared for each species between areas using Analysis of Variance (ANOVA).

### Ethical approval

Collection procedures were approved by the “Ministério do Meio Ambiente do Brasil” - (SISBIO: license number 15,237).

## Results

The phlebotomine fauna was represented by 15 species. Table 1 shows the total number of phlebotomines collected by study area, and also the Shannon diversity index (H) and the evenness of Pielou (J). The monthly samples yielded 3,365 specimens of phlebotomines, of which 1,900 were males (56.46 %) and 1,465 were females (43.54 %).

**Table 1:** Species of phlebotomines collected during March 2013 to February 2014 in the municipality of Várzea da Palma, Minas Gerais, Brasil, by study area and sex and with their respective diversity (H) and evenness (J) indicies

	Urban area		Transitional area		Wild area						Total (%)
Taxonomic category	A		T1		T2		T3		T4		
	F	M	F	M	F	M	F	M	F	M	
*Brumptomyia avellari*	13	15	33	25	48	102	9	10	22	40	317 (9.42)
*Evandromyia evandroi*	6	8	49	9	141	19	29	4	97	16	378 (11.23)
*Evandromyia lenti*	16	35	6	36	27	195	9	35	28	140	527 (15.66)
*Evandromyia sallesi*	52	27	5	0	15	1	12	12	14	5	143 (4.25)
*Evandromyia termitophila*	1	3	2	0	12	3	4	0	14	1	40 (1.19)
*Evandromyia walkeri*	2	8	0	2	2	2	1	0	1	1	19 (0.56)
*Lutzomyia longipalpis*	101	435	2	19	1	12	0	4	2	5	581 (17.27)
*Micropygomyia quinquefer*	14	16	0	0	0	0	0	0	0	1	31 (0.92)
*Nyssomyia intermedia*	589	539	10	17	1	4	4	2	9	0	1.175 (34.92)
*Nyssomyia neivai*	52	76	2	3	0	2	0	1	0	1	137 (4.07)
*Nyssomyia whitmani*	0	0	1	0	0	0	0	0	0	0	1 (0.03)
*Pintomya pessoai*	0	0	1	0	1	0	0	0	0	0	2 (0.06)
*Psathyromyia lutziana*	0	1	2	0	0	1	0	0	0	0	4 (0.12)
*Psathyromyia bigeniculata*	1	1	0	0	0	0	0	0	1	1	4 (0.12)
*Sciopemyia sordellii*	1	0	0	2	0	2	0	1	0	0	6 (0.18)
Total (%)	2.012 (59.79)		226 (6.72)		1.127 (33.49)						3.365 (100)
Diversity Index (H)	1.2823		1.8694		1.5572						1.8529
Evenness Index (J)	0.4999		0.7288		0.5901						0.6842

A = urban area; T1 = transitional area; T2-T4 = wild area

The urban area had the greatest number of phlebotomines collected with 2,012 individuals, followed by the forest area with 1,127, and the transitional area with 226. By standardizing the number of phlebotomines collected in the different areas, due to differences in sampling effort, the capture success was also highest in the urban area (27.94 phlebotomine/trap), followed by the forest area (5.22), and lastly the transitional area (3.14).

Considering all of the sampled areas, the species most frequently trapped was *Ny. intermedia* with 34.92 % of the total number of phlebotomines collected, followed by *Lu. longipalpis* 17.27 %, *Ev. lenti* 15.66 %, *Ev. evandroi* 11.23 %, *Br. avellari* 9.42 % and *Ny. neivai* and *Ev*. s*allesi,* both with 4.07 %.

When testing these seven most prevalent species using ANOVA, four (*Ev. sallesi*, *Lu. longipalpis*, *Ny. intermedia* and *Ny. neivai*) showed significant differences in abundance among study sites (p < 0.05), with greater abundance in the urban area (Table 2).

**Table 2:** Main species of phlebotomines collected in urban, transition, and wild areas of the district of Barra do Guaicui, Minas Gerais, Brasil, during March 2013 to February 2014

	Study area						
	Urban		Transitional		Wild		
Species	N	%	N	%	N	%	Total
*Brumptomyia avellari*	28	0.86	58	1.78	231	7.09	317
*Evandromyia evandroi*	14	0.43	58	1.78	306	9.39	378
*Evandromyia lenti*	51	1.57	42	1.29	434	13.32	527
*Evandromyia sallesi*	79	2.42^a^	5	0.15^b^	59	1.81^b^	143
*Lutzomyia longipalpis*	536	16.45^a^	21	0.64^b^	24	0.74^b^	581
*Nyssomyia intermedia*	1128	34.62^a^	27	0.83^b^	20	0.61^b^	1175
*Nyssomyia neivai*	128	3.93^a^	5	0.15^b^	4	0.12^b^	137

^a, b^Different letters indicate significant differences between the columns; p < 0.05

Species richness of phlebotomines was approximately equal in all of the areas, with 14 species being collected in the wild area and 13 in both the urban and transitional areas. The transitional area had the highest diversity and evenness values, 1.869 and 0.728, respectively, whereas the urban area had the lowest, 1.282 and 0.499, respectively (Table 1).

The species *Ny. whitmani* was encountered exclusively in the transitional area whereas *Mi. quinquefer* and *Pa. bigeniculata* were not collected in this area. *Pintomyia pessoai* was collected only in the transitional and wild areas.

**Fig. 3:**
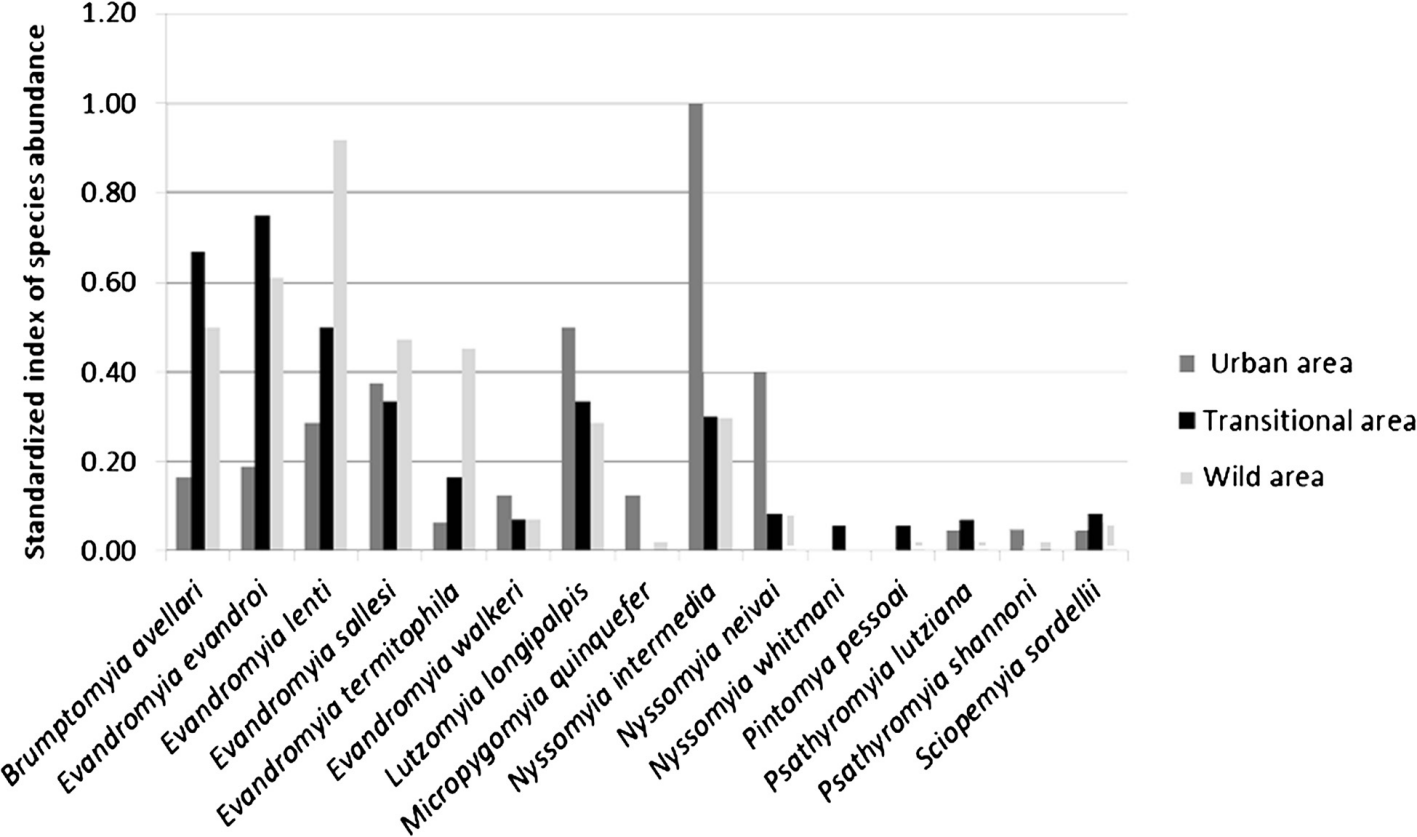
Standardized Abundance Indexes for the species collected utilizing automatic light traps in the district of Barra do Guaicuí, Minas Gerais, Brasil, during March 2013 to February 2014

In the urban area, *Ny. intermedia* was the most abundant species with SISA = 1, followed by *Lu. longipalpis* (SISA = 0.50), *Ny. neivai* (0.40) and *Ev. sallesi* (0.38). In the transitional area, *Ev. evandroi* was the most abundant (0.75) followed by *Br. avellari* (0.67) and *Ev. lenti* (0.50). This latter species was the most abundant species in the wild area with SISA = 0.92, followed by *Ev. evandroi* (SISA = 0.61). *Nyssomyia intermedia*, *Lu. longipalpis* and *Ev. sallesi* were well distributed in the transitional and wild areas with very close SISA values (Fig 3).

## Discussion

The presence of 15 species of phlebotomines in the collections made in the district of Barra do Guaicuí matches previous findings for the northern region of the state of Minas Gerais [18, 19].

The phlebotomine fauna found in the district of Barra do Guaicui showed to be diverse and predominantly comprised of important vector species, such as *Lu. longipalpis* and *Ny. intermedia* that are involved in disseminating the etiologic agents of VL and TL, respectively [20, 21]. In addition to these species, *Ev. lenti*, *Ev. sallesi*, *Ny. neivai* and *Ny. whitmani* deserve attention because they have been found naturally infected and some may be involved in the wild and/or urban cycle of leishmaniases [21–24]. This faunistic composition is typical of modified environments near secondary forests, as seen in other areas of southeastern Brazil [25].

The species with the highest density was *Ny. intermedia*, followed by *Lu. longipalpis*. These results are different from those observed in other studies conducted in northern Minas Gerais, where *Lu. longipalpis* was identified as the predominant species [19, 26, 27].

It is understood that there is a correlation between the density of *Lu. longipalpis* and peridomicile conditions and this species is frequently associated with the presence of domestic animals [28, 29]. This behavioral characteristic was evident in the present study, since there were a greater number of individuals of this species in samples from peridomicile locations in urban area, with the presence of breeding animals.

The species *Ny. intermedia* was predominant in the urban area. This species is considered of great importance in the transmission of *Leishmania braziliensis* in southeastern Brazil [21, 30]. In Minas Gerais, *Ny. intermedia* is prevalent or quite abundant in endemic areas of TL. Gontijo *et al.,* in a study conducted in a TL outbreak in Vale do Jequitinhonha, Minas Gerais, Brazil described the prevalence of *Ny. intermedia* in the region and its preference for environments with a great degree of anthropic modification [31]. According to the high population density encountered in the district of Barra do Guaicuí, *Ny. intermedia* may be participating in the transmission cycle of *Leishmania* species along with *Lu. longipalpis*.

When comparing the number of specimens in each studied environment greater capture success is evident in the urban environment. This capture success can be explained by the presence of domestic animals near the places where the traps were exposed. However, with regard to diversity, the opposite was observed, with the transitional area showing the greatest diversity, followed by the wild environment and lastly the urban area. The same trend was observed for the evenness index, which remained high in places with the greatest diversity of species, but low in the urban area, where *Ny. intermedia* showed absolute dominance.

The fact that the urban area possesses some rural characteristics, such as livestock and subsistence crops, probably led to it having the largest number of insects collected. Some authors emphasize the importance of pigsties or chicken coops as risk factors because they serve as locations for the creation and maintenance of a high density of phlebotomines [32–35]. The greatest diversity (H) in the transitional area can also be explained by the fact, that it is located between the forest and the urban environment.

The analysis of species abundance among the three areas showed different results in each of them, with absolute predominance of *Ny. intermedia* in the urban area, *Ev. evandroi* in the transition area and *Ev. lenti* in the wild area. Galati *et al.,* also reported *Ny. intermedia* to have the highest SISA ranking in the Província Espeleológica do Vale do Ribeira, state of São Paulo, Brazil [36]. The populations of the species collected in the transitional and wild areas seem better adapted to forest environments, a fact also reported by other authors [37–39], unlike those species that predominate in urban areas that are better adapted to anthropic environments or forest edge [40–42]. Beyond these above mentioned species, it is important to highlight the presence of *Lu. longipalpis* in urban areas, *Br. avellari* and *Ev. lenti* in the transition area and *Ev. evandroi* and *Br. avellari* in the wild area, all of these species with SISA above 0.50.

Thus, whereas both areas have similar phlebotomine faunas, differing only in a few species, the situations with regard to the abundance of vectors of *Leishmania* spp. (*Ny. intermedia*, *Ny. neivai*, *Ev. sallesi* and *Lu. longipalpis*) are completely different. It was possible to confirm the pattern of adaptation of these species to peridomicile areas and the modified environment of the municipality of Várzea da Palma. The predominance of some species in urban environments is a relevant factor for the transmission of *Leishmania* species and has been mentioned in other faunal studies of phlebotomines [37, 43–45].

Although *Ny. whitmani* is represented by only one specimen in the district of Barra do Guaicuí, this finding requires special attention because of its implication as a potential vector of *L. braziliensis* in the northeast region of Brazil [20, 45].

Interesting to highlight the greater abundance of *Ny. intermedia* compared to *Lu. longipalpis* in the urban area of Guaicuí bar district. This finding is different from that reported in studies in urban areas of Minas Gerais, where the predominance of *Lu. longipalpis* is always registered [46–48]. *Nyssomyia intermedia* was found naturally infected by *Leishmania infantum* in Minas Gerais State and other regions of Brazil [22, 49, 50] and the highest population density in the urban area, which can be an indication that in this area this species may be playing a role in the transmission cycle of the *L. infantum*.

## Conclusion

In a year of sand flies collection it was possible to demonstrate the distribution, richness and abundance of species in three different ecotypes. The distribution of the collected species showed distinct profiles between the environments, highlighting the potential risk of transmission of leishmaniasis in the urban environment where it was observed the highest population density and abundance of important vector species of *Leishmania*. Despite the different ecological characteristics between environments the richness of species was very similar between the areas. The results of this study contribute to understanding the gradual urbanization of the species of sand flies found in the state of Minas Gerais. Furthermore it demonstrates that the municipality of Várzea da Palma has several important characteristics for the expansion of visceral and cutaneous leishmaniasis.
